# Novel Mutations of RPGR in Chinese Retinitis Pigmentosa Patients and the Genotype-Phenotype Correlation

**DOI:** 10.1371/journal.pone.0085752

**Published:** 2014-01-15

**Authors:** Liping Yang, Xiaobei Yin, Lina Feng, Debo You, Lemeng Wu, Ningning Chen, Aijun Li, Genlin Li, Zhizhong Ma

**Affiliations:** 1 Department of Ophthalmology, Peking University Third Hospital, Key Laboratory of Vision Loss and Restoration, Ministry of Education, Beijing, P. R. China; 2 Beijing Tongren Eye Center, Beijing Tongren Hospital, Capital Medical University, Beijing, P. R. China; Innsbruck Medical University, Austria

## Abstract

X-linked Retinitis Pigmentosa (XLRP) accounts for 10–20% of all RP cases, and represents the most severe subtype of this disease. Mutations in the Retinitis Pigmentosa GTPase Regulator (RPGR) gene are the most common causes of XLRP, accounting for over 70–75% of all XLRP cases. In this work, we analyzed all the exons of RPGR gene with Sanger sequencing in seven Chinese XLRP families, two of these with a provisional diagnosis of adRP but without male-to-male transmission. Three novel deletions (c.2233_34delAG; c.2236_37delGA and c.2403_04delAG) and two known nonsense mutations (c.851C→G and c.2260G→T) were identified in five families. Two novel deletions (c.2233_34delAG and c.2236_37delGA) resulted in the same frame shift (p.E746RfsX22), created similar phenotype in Family 3 and 4. The novel deletion (c.2403_04delAG; p.E802GfsX31) resulted in both XLRP and x-linked cone-rod dystrophy within the male patients of family 5, which suggested the presence of either genetic or environmental modifiers, or both, play a substantial role in disease expression. Genotype-phenotype correlation analysis suggested that (1) both patients and female carriers with mutation in Exon 8 (Family 1) manifest more severe disease than did those with ORF15 mutations (Family 2&3&4); (2) mutation close to downstream of ORF15 (Family 5) demonstrate the early preferential loss of cone function with moderate loss of rod function.

## Introduction

Retinitis pigmentosa (RP) is an inherited retinal degeneration that affects approximately one in 3500 individuals, with an estimated total of 1.5 million patients worldwide [1]. RP is caused by progressive loss of rod and cone photoreceptors. Typical symptoms include night blindness followed by decreasing visual fields, leading to tunnel vision and eventually blindness. Clinical hallmarks of RP include bone-spicule deposits, attenuated retinal blood vessels, optic disc pallor, visual field loss, and abnormal, diminished or non-recordable electroretinographic responses (ERG).

RP can be inherited in an autosomal dominant (adRP), autosomal recessive (arRP), or X-linked (XLRP) manner, with rare digenic and mitochondrial forms [2]. To date, more than 53 genes are known to cause RP (https://sph.uth.edu/retnet/disease.htm). XLRP accounts for 10–20% of all RP cases [3], and represents the most severe subtype of this disease. The earliest clinical manifestation of XLRP in males is night blindness, with onset in the first decade, progressing to a reduction in the visual fields in the second decade, a reduction in visual acuity by the third to fourth decade, and severe visual loss (<20/200) by age 40 years [4]. Until now, six loci (RP2, RP3, RP6, RP23, RP24, and RP34) on the X-chromosome have been mapped (https://sph.uth.edu/retnet/disease.htm), with two genes (Retinitis Pigmentosa GTPase Regulator [RPGR] and RP2) were identified. Mutations in the RPGR gene are the most common causes of XLRP, accounting for over 70–75% of all XLRP cases [5]–[7]. Although XLRP is thought to affect male subjects only, many documented cases of RPGR cause disease in carrier female subjects, which giving an impression of occurrence in sequential generations simulating Mendelian dominant transmission [6]. It has been reported that RPGR carrier female subjects exhibit a range of phenotypes that can vary from asymptomatic to severe retinal disease similar to male subjects [8]–[10]. The presence of “affected” or, at least, partially manifesting female subjects with an absence of male-to-male transmission in a pedigree may lead to misinterpretation [6], which makes the genetic diagnosis difficult and complex.

RPGR is predominantly a cilia-centrosomal protein [11]–[12], and is suggested to regulate cilia function and facilitate trafficking of proteins along the photoreceptor cilium [13]. Approximately 60% of disease causing mutations in RPGR is found in ORF15, an alternatively spliced exon including exon 15 and extending into intron 15 [14]. ORF15 is highly repetitive and purine-rich, which promotes polymerase arrest and slipped strand mispairing, thus making ORF15 a hotspot for mutations [3], [14].

The goal of this study was to identify the disease causing mutations in seven Chinese XLRP families, two of these with a provisional diagnosis of adRP but without male-to-male transmission, and to characterize the phenotypic manifestation associated with the mutation.

## Materials and Methods

### Ethics Statement

All experiments involving DNA of the patients and their relatives were approved by Peking University Third Hospital Medical Ethics Committee (No. 2012093). Written informed consent was obtained from all participants or guardians on behalf of the minors/children participants, and the ethics committees approve this consent procedure.

### Patients

All patients and controls were identified from Peking University Eye Center and Beijing Tongren Eye Center. The diagnosis of RP was based on night blindness beginning in early childhood, progressive loss of peripheral vision, decreasing visual acuity with age, waxy pale discs, retinal arteriolar attenuation, scattered bone-spicule pigmentation in the mid-peripheral retina, and reduced rod and cone function on electroretinography (ERG). Medical and ophthalmic histories were obtained, and ophthalmological examination was carried out. One hundred of general healthy individuals from the Chinese Han ethnic population were recruited to serve as controls. All procedures used in this study conformed to the tenets of the Declaration of Helsinki.

### Mutation Screening

Blood samples were collected and genomic DNA was extracted by standard protocols (D2492 Blood DNA Maxi Kit, Omega Bio-Tek Inc, GA, USA). All the exons (exons 1–19) and exon-intron boundaries of RPGR (NG_009553.1; NM_000328) were amplified with High fidelity Taq polymerase (Invitrogen, Grand Island, NY, USA) using Touchdown PCR with primers listed in Table S1. Touchdown PCR amplification consisted of a denaturizing step at 95°C for 5 minutes, followed by 35 cycles of amplification (at 95°C for 30 seconds, at 64∼57°C for 30 seconds starting from 64°C with decresing by 0.5°C every cycle for 14 cycles until remaining at 57°C for 21 cycles, and at 72°C for 40 seconds), and a final extension at 72°C for 10 minutes as described previously [15]. Purified PCR products were sequenced using the ABI BigDye Terminator cycle sequencing kit on ABI 3130XL genetic analyzer (ABI Applied Biosystem, Foster City, CA, USA). Amplification of the RPGR exon ORF15 (NG_009553.1; NM_001034853) was carried out by using primers located outside the repetitive stretch as described previously [5]. The forward primer is 5′-CAGAGATCCTATCAGATGACC-3′, and the reverse primer is 5′-TGTCTGACTGGCCATAATCG-3′, with PCR product 1630 bp. PCR products were sequenced using four reported reverse primers [5]. Sequencing results were analyzed with Sequencher (Gene Codes Corporation, Ann Arbor, MI, USA); the identified mutations were further evaluated for segregation in available family members and 100 controls by sequencing. Primer pairs for individual exons were designed using the Primer program (http://www.yeastgenome.org/cgi-bin/web-primer).

## Results and Discussion

Seven families, a total of 101 individuals were recruited in this study, and five mutations in RPGR gene were detected in five families. These mutations included two nonsense mutations (c.851C→G; p.S284X identified in Family 1 and c.2260G→T; p.E754X identified in Family 2) and three novel deletions (c.2233_34delAG identified in Family 3; c.2236_37delGA identified in Family 4; and c.2403_04delAG identified in Family 5). Three deletions resulted in frame shifts, creating truncated proteins with a premature stop codon (p.E746RfsX22 and p.E802GfsX31). It is noteworthy that both c.2233_34delAG (Family 3) and c.2236_37delGA (Family 4) led to the same protein change p.E746RfsX22, creating similar phenotype in Family 3 and 4. The novel deletion (c.2403_04delAG; p.E802GfsX31) in Family 5 resulted in varying degrees of expressivity and different diagnoses within the family. These mutations co-segregated in affected males and obligate female carriers, but were absent in 100 matched controls.

**Figure 1:**
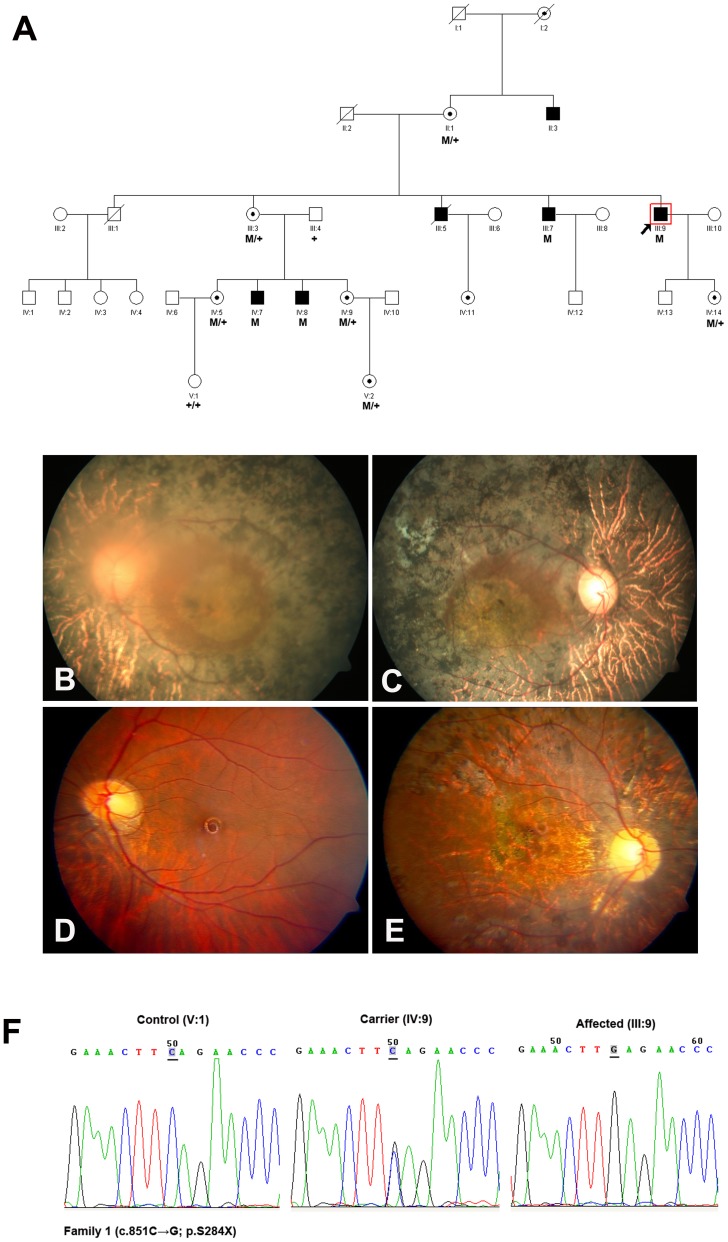
Family 1 with nonsense mutation c.851C>G (p.S284X) in RPGR gene. **A:** The pedigree of Family 1. Filled symbols represent affected, unfilled unaffected, dotted carrier. Square signify male, circles females. Arrow marks the index patient. M refers to the mutant allele, and + means normal allele. **B**–**C:** Fundus photographs of the 57-year-old proband (III:9) manifested typical retinitis pigmentosa changes. **D**–**E:** Fundus photographs of the carrier (IV:9) showed pigment deposits, pallor of the disc and RPE degeneration in right eye. **F:** Representative sequence chromatograms for a normal individual (left), a female mutation carrier (middle) and a male patient (right).

To date, nearly 350 different RPGR gene mutations have been found in families with XLRP (Human Gene Mutation Database; http://www.hgmd.cf.ac.uk), and different types of RPGR mutation caused different phenotypes [7], [16], [17]. Previous study from Ferreira PA demonstrated that RPGR mutations bearing premature termination codons (PTCs) before the last exon behaved as null allele, resulted in haploinsufficiency as their corresponding mRNA was degraded by nonsense-mediated mRNA decay (NMD) [18]. In this study we further described a nonsense mutation (c.851C>G; p.S284X), firstly found in USA with moderate clinical manifestation, caused severe visual abnormality in patients in Family 1 (Figure 1A–F). The obligatory female carriers in this family manifested high myopia accompanied by severe retinal and visual function abnormalities in one eye. The fundoscopic and functional changes were beyond those usually seen in carriers of XLRP, led to the diagnosis of a semi-dominant pattern of inheritance. The N-terminal part of RPGR contains a domain homologous to the regulator of chromosome condensation 1 (RCC1), which contains seven-bladed propeller repeats and regulates the GTPase RAN. All currently known direct RPGR interactions (such as with RPGR-interacting protein 1 and structural maintenance of chromosome protein 1 or 3) were occured via the RCC1-like domain [19]. The nonsense mutation (c.851C>G; p. S284X) in Family 1 happened in Exon 8 of RPGR, resulted in PTC before the last exon, led to the disruption of RCC1-like domain (NP_001030025), thus the functional loss of RPGR with other interaction partners was the most probably reason for Family 1.

**Figure 2:**
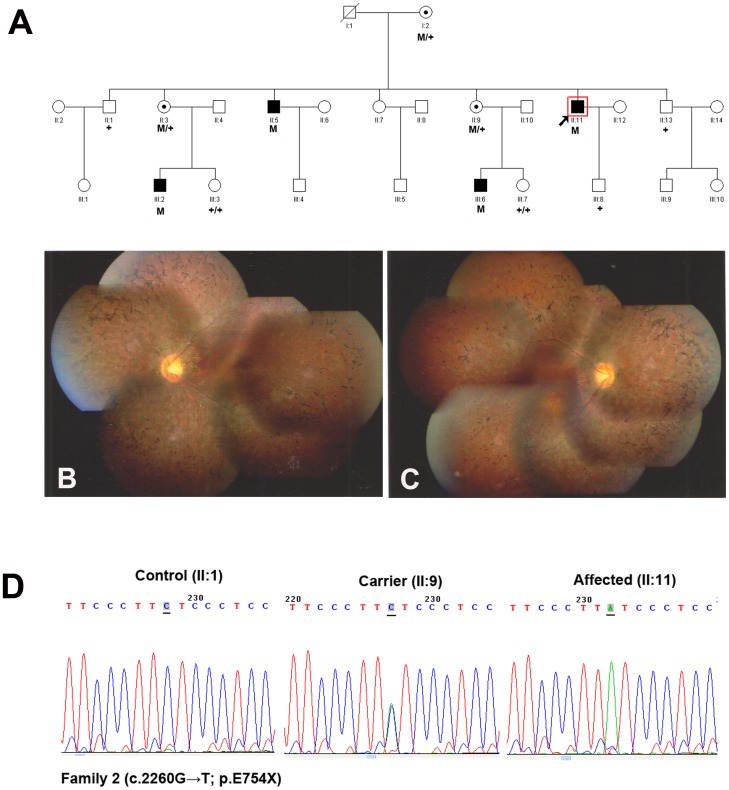
Family 2 with nonsense mutation c.2260G>T (p.E754X) in RPGR gene. **A:** The pedigree of Family 2. Filled symbols represent affected, unfilled unaffected, dotted carrier. Square signify male, circles females. Arrow marks the index patient. M refers to the mutant allele, and + means normal allele. **B**–**C:** Fundus photographs of the 37-year-old proband (II:11) manifested bone spicule like pigmentation in the middle and periphery retina, attenuated arterioles and RPE degeneration. **D:** Representative sequence chromatograms for a normal individual (left), a female mutation carrier (middle) and a male patient (right).

**Figure 3:**
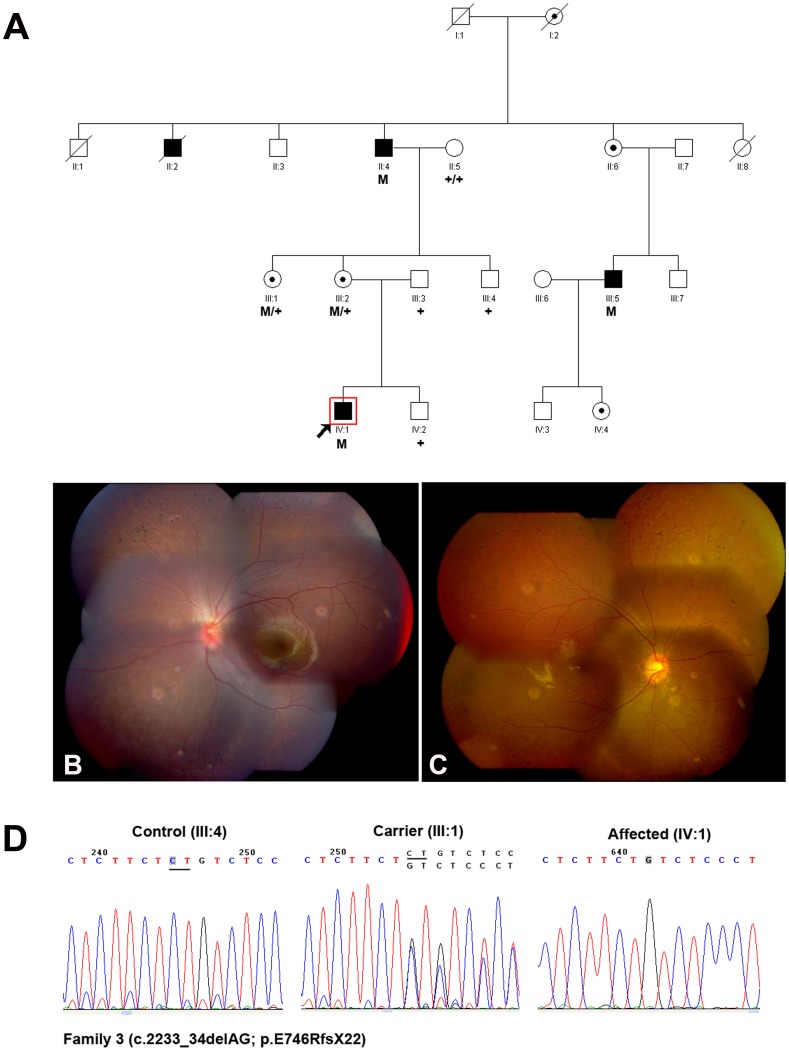
Family 3 with a novel deletion c.2233_34delAG (p.E746RfsX22) in ORF15 of RPGR gene. **A:** The pedigree of Family 3. Filled symbols represent affected, unfilled unaffected, dotted carrier. Square signify male, circles females. Arrow marks the index patient. M refers to the mutant allele, and + means normal allele. **B**–**C:** Fundus photographs of the 8-year-old proband (IV:1) showed bone spicule pigment deposits at the periphery retina. **D:** Representative sequence chromatograms for a normal individual (left), a female mutation carrier (middle) and a male patient (right).

**Figure 4:**
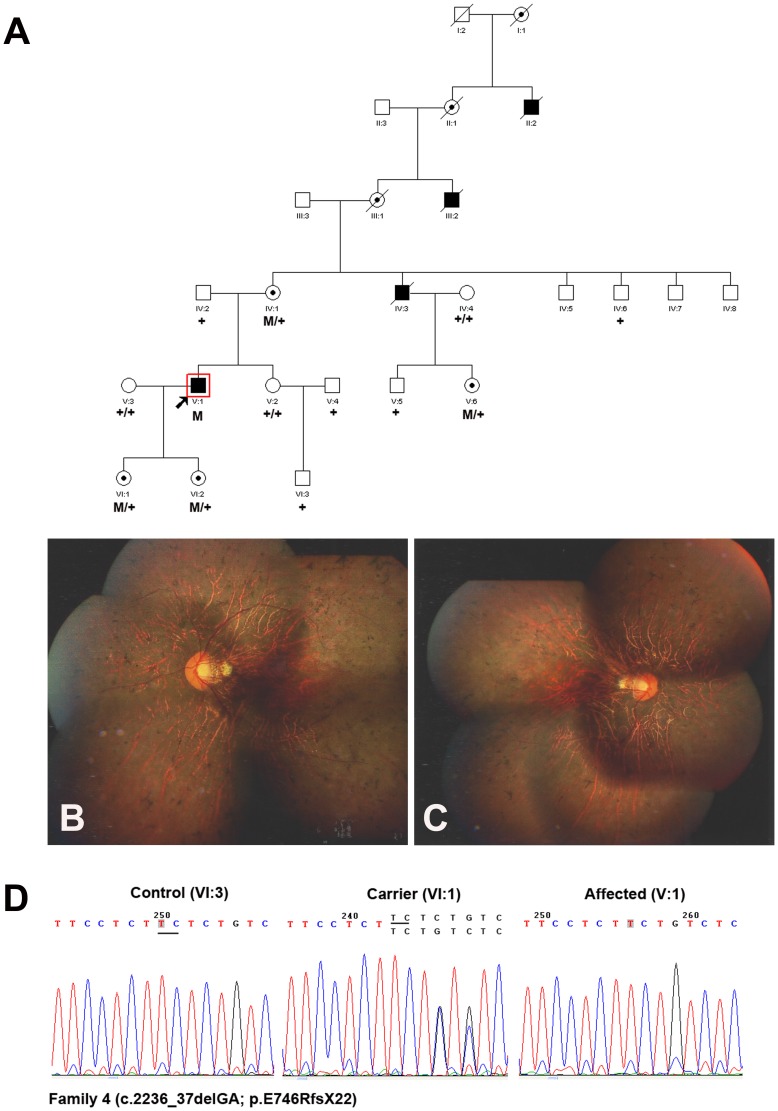
Family 4 with a novel deletion c.2236_37delGA (p.E746RfsX22) in ORF15 of RPGR gene. **A:** The pedigree of Family 4. Filled symbols represent affected, unfilled unaffected, dotted carrier. Square signify male, circles females. Arrow marks the index patient. M refers to the mutant allele, and + means normal allele. **B**–**C:** Fundus photographs of the 42-year-old proband (V:1) showed bone spicules like pigmentation, attenuated blood vessels, waxy pallor of the disc and RPE degeneration. **D:** Representative sequence chromatograms for a normal individual (left), a female mutation carrier (middle) and a male patient (right).

The photoreceptor-specific RPGR mRNA containing the alternative ORF15 (RPGR^ORF15^) encodes 1152 amino acids, which plays a major role in the transport mechanisms within the connecting cilium (CC) [20]. In addition to exons 1–14 that it shares with RPGR^ex1-19^, it contains a 567 amino acids encoding terminal exon, rich in glutamic acid alternating with glycine as a repetitive motif that consists of an imperfect repeat of EEEGEGEGE in the human [21]. It is important to note that mutations in the ORF15 exon will not lead to a mechanism known as NMD, as this exon is the terminal exon of the gene, it is thought to be translated into a truncated protein. In this study, a nonsense mutation in the ORF15 exon (c.2260G→T; p.E754X) was identified in Family 2 (Figure 2 A–D), and two novel deletions (c.2233_34delAG and c.2236_37delGA) were identified in Family 3 (Figure 3A–D) and Family 4 (Figure 4A–D) respectively. The two deletions resulted in frame shifts, created the same truncated protein with a premature stop codon (p.E746RfsX22). Both the nonsense mutation and the deletion happened at the beginning of glutamic acid repetitive region of ORF15 exon. The shortened protein will not contain the GE motif, and the 2bp shift generated a predominant Glycin and Arginin rich motif. The newly generated chains contained mostly basic amino acids, thus changed some of the major biochemical properties of the protein [14], [22]. Transgenic studies demonstrated certain truncated forms of RPGR could behave as a dominant, gain-of-function mutant, which caused more rapid photoreceptor degeneration than that in the RPGR null mutant [11]. Inconsistent with this, not only in hemizygous males but also in heterozygous female, the phenotype demonstrated in Family 3&4 (carried the frame shift mutation) was comparable to that in Family 2 (carried the nonsense mutation).

**Figure 5:**
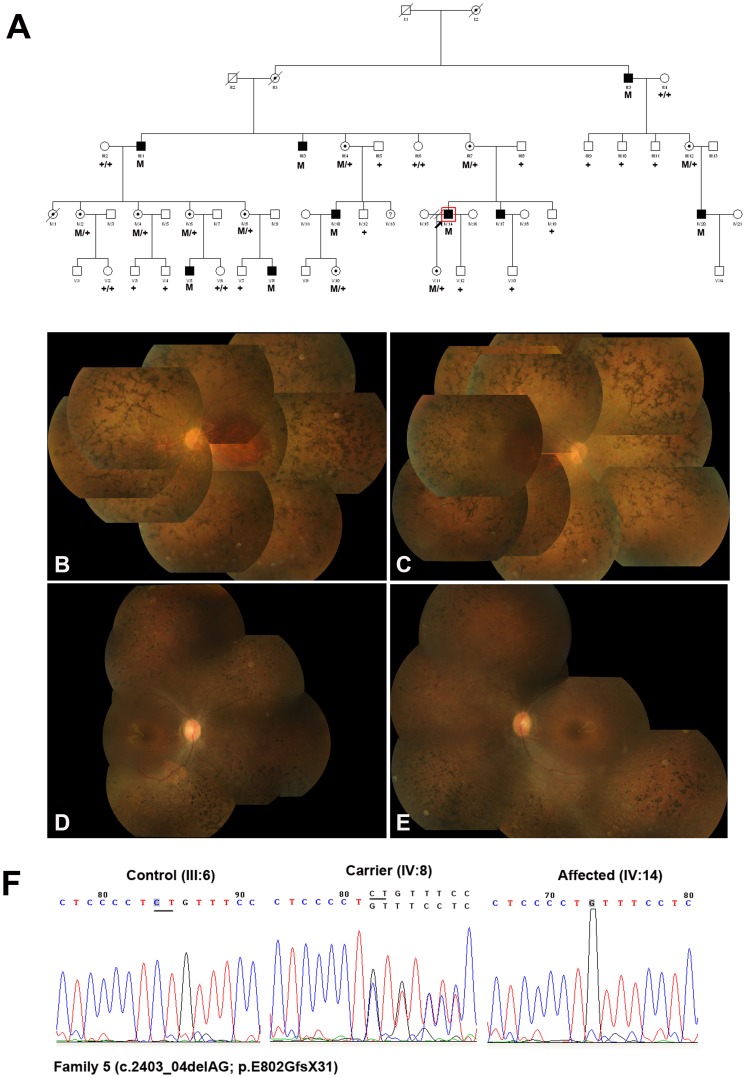
Family 5 with a novel deletion c.2403_04delAG (p.E802GfsX31) in ORF15 of RPGR gene. **A:** The pedigree of Family 5. Filled symbols represent affected, unfilled unaffected, dotted carrier. Square signify male, circles females. Arrow marks the index patient. M refers to the mutant allele, and + means normal allele. **B**–**C:** Fundus photographs of the 40-year-old proband (IV:14) showed characteristic bone spicule pigment deposits, attenuation of retinal arterioles and RPE degeneration. **D**–**E:** Fundus photographs of the 12-year-old patient (V:5) demonstrated pigment deposits in the middle and periphery retina, with white dots scattered in the pigment deposits. **F:** Representative sequence chromatograms for a normal individual (left), a female mutation carrier (middle) and a male patient (right).

It is interesting that some mutations in ORF15 caused typical RP, but others caused cone dystrophy, cone-rod dystrophy or atrophic macular degeneration [6], [23]–[26]. In addition, there have been multiple reports of diagnoses of both XLRP and cone-rod dystrophy within the same family [27]–[29]. In this study, a novel deletion (c.2403_04delAG; p.E802GfsX31) was identified in Family 5 (Figure 5A–F). The deletion happened at the one third of GE motif, and the same mutation resulted in varying degrees of clinical severity and different diagnoses within the male patients. Fundus examinations in the 40-year-old proband (IV:14) showed characteristic RP phenotype such as bone spicule pigment deposits, attenuation of retinal arterioles and RPE degeneration (Figure 5B–C). Fundus examination of another 12-year-old patient (V:5) demonstrated pigment deposits in the middle and periphery retina, with white dots scattered in the pigment deposits. The ERG results demonstrated that both rod and cone cells were affected, led to the diagnosis of cone-rod dystrophy. Most of the obligatory female carriers had high myopia with a refractive error ranging from −6.00D to −20.00D and severe visual abnormalities. The fundoscopic and functional changes were beyond those usually seen in carriers of XLRP, led to the diagnosis of a semi-dominant pattern of inheritance at first. The variable expressivity in the male patients of Family 5 supported the presence of either genetic or environmental modifiers, or both, play a substantial role in disease expression. But the exact mechanism of which modifier played a significant role in this pedigree was still unclear, which needs to be further investigated.

A correlation between the localization of the RPGR mutation and the resulting phenotype seems likely. Cross-sectional analyses suggested that patients with mutations in exons 1 to 14 were more likely to have severe disease than did patients with ORF15 mutations [16]–[17]. Consistent with this, our study demonstrated that both patients and female carriers with mutation in Exon 8 (Family 1) manifested more severe disease than did most of those with ORF15 mutations (Family 2&3&4). Shu X et al. assumed that if the mutation is located in direction of the 3′ end of ORF15 and thereby produces a shorter altered protein sequence, the RP phenotype would be milder possibly due to better maintenance of rod function [22]. In contrast, previous reports from Sharon D et al. [16] demonstrated that the mutations close to down-stream of ORF15 implicated the early preferential loss of cone function. Our results that most of patients carried the down-stream of ORF15 mutation (Family 5) manifested cone-rod dystrophy, and all the patients carried the relative up-stream of ORF15 mutation (Family 2&3&4) demonstrated characteristic RP supported the idea that mutation close to downstream of ORF15 demonstrated the early preferential loss of cone function.

In conclusion, we identified 5 mutations (3 were novel mutations) in five Chinese families with X-linked retinitis pigmentosa and documented the clinical manifestations. Our findings broaden the spectrum of RPGR mutations causing XLRP and phenotypic spectrum of the disease in Chinese families, which will be useful for genetic consultation and genetic diagnosis in the future.

## Supporting Information

Table S1 **Primers used for RPGR amplification and sequencing.**
(DOCX)
