# Supplementary material for: Novel Mutations of RPGR in Chinese Retinitis Pigmentosa Patients and the Genotype-Phenotype Correlation
Source: PLoS One. 2014 Jan 15;9(1):e85752. doi: 10.1371/journal.pone.0085752 (PMC3893273; doi:10.1371/journal.pone.0085752)
Supplement: Table S1 — Primers used for RPGR amplification and sequencing. (DOCX) [file pone.0085752.s001.docx]

**Table S1.** Primers used for RPGR amplification and sequencing.

| S1. Primers used for RPGR amplification and sequencing. | | | |
| --- | --- | --- | --- |
| Primer | **Forward** | **Reverse** | **Product (bp)** |
| RPGR-exon-1 | AACCGTCCTCTACAGCCTC | CTCTTTCCCGTTCTCCCC | 285 |
| RPGR-exon-2&3 | GGAAGGCTTAAACATTGCCA | TGGGGGATATTCAAATGCAA | 1069 |
| RPGR-exon-4 | CTGAAAACCTCTGGTTTGCT | TGCAAAGGCAAACGTGTACT | 374 |
| RPGR-exon-5 | CCTTGCTTGTTTTGCTTTAT | TTCGGTTTACTGAGTTGGC | 409 |
| RPGR-exon-6 | CAATCAGGCTGTTCTGTGTTC | CTGAGAAAGTCCCACCAAAG | 634 |
| RPGR-exon-7 | ATCTCAAGAAAGGTCAAATGTA | TAATAAAATGGTGGTCGCC | 561 |
| RPGR-exon-8 | TTTTCCCCAGAGGCACTTA | TTCTGACATCATCGGCCTAT | 517 |
| RPGR-exon-9 | TTACATGCAGGACCACAGAGA | AAAGGAAGAGGCTAAAGGAGG | 512 |
| RPGR-exon-10 | GATTCACCAAGCCAGTCTGT | AGCACCATTGTCTATATGCAA | 563 |
| RPGR-exon-11 | AATGTTGTGGAGTGTTGGCA | AGGATATTCCCGGATTTGAG | 452 |
| RPGR-exon-12&13 | TCAATTTCCCTGACATGAGG | CCAAAGAGCAAATTTCAGCA | 898 |
| RPGR-exon-14 | GAGAGTGGCACAAATGATCCT | TGTCCTCCATCACTTTCCTTT | 502 |
| RPGR-exon-15a | TGATGAAGTGGAAACTGACC | CCTCATCTTGCCAGTGTTCT | 395 |
| RPGR-exon-16 | CAGCAATATCAAATCCCTCG | TTCACAAATAAGCCAAAGCC | 424 |
| RPGR-exon-17 | GGACATTTGCAATCAGTCAG | ATAGCTTGTTATGAGGATCATG | 397 |
| RPGR-exon-18 | GGGTGAGCTCTTTTCGTATTA | CAAATGCTAACCAAAGGAAAG | 389 |
| RPGR-exon-19 | ATCCTGACTGCCTTTTGGT | GCCACAACACTTTAGGGAGA | 624 |
|  |  |  |  |
| RPGR ORF15 PCR primers | CAGAGATCCTATCAGATGACC | TGTCTGACTGGCCATAATCG | 1630 |
| RPGR ORF15 reverse sequence primer 1 | TGTCTGACTGGCCATAATCG | |  |
| RPGR ORF15 reverse sequence primer 2 | GTTTGCCATATTTCACAGATCC | |  |
| RPGR ORF15 reverse sequence primer 3 | TCCTTCCTCCTCTTCCCCCTCCCA | |  |
| RPGR ORF15 reverse sequence primer 4 | CCTTCCTCCTCTTCCCCCTCA | |  |
